# Cytomegalovirus Lymphadenitis Mimicking Hodgkin Lymphoma Relapse

**DOI:** 10.7759/cureus.77649

**Published:** 2025-01-19

**Authors:** Belit M Cagdas, Ismail Kaygisiz, Enver Yarıkkaya, Özgür YIGIT

**Affiliations:** 1 Otolaryngology - Head and Neck Surgery, University of Health Sciences Istanbul Training and Research Hospital, Istanbul, TUR; 2 Pathology, University of Health Sciences Istanbul Training and Research Hospital, Istanbul, TUR

**Keywords:** cytomegalovirus lymphadenitis, differential diagnosis, histopathology, hodgkin lymphoma, infectious lymphadenitis

## Abstract

Cytomegalovirus (CMV) reactivation is a known complication in patients with hematological cancers and those who undergo stem cell transplants, often resembling cancer relapse. CMV lymphadenitis, an infection localized to the lymph nodes, can be difficult to distinguish from lymphoma recurrence based on clinical and radiological findings alone. We report a case of a 40-year-old male treated for Hodgkin lymphoma who presented with cervical lymphadenopathy nine months after treatment. PET-CT showed a hypermetabolic lesion, suggesting relapse, but a biopsy confirmed CMV lymphadenitis. Systemic CMV infection was excluded based on negative PCR results and normal thoracic imaging. This report highlights the importance of histopathological confirmation in patients with a history of lymphoma, as CMV lymphadenitis can mimic relapse. Accurate diagnosis prevents unnecessary treatments and avoids misinterpretation of imaging findings. In isolated lymph node cases without systemic symptoms, observation may be sufficient as the condition can resolve spontaneously. This underscores the need for careful evaluation to ensure correct diagnosis and appropriate management.

## Introduction

Cytomegalovirus (CMV) reactivation is one of the most common and significant complications in patients with hematological malignancies undergoing allogeneic stem cell transplantation. However, the critical nature of this condition has not been adequately assessed in other hematological diseases. This is primarily due to the lower incidence of CMV reactivation in non-transplant hematological clinical settings. In recent years, the use of pleiotropic immunosuppressive chemotherapy, particularly in lymphoproliferative disorders, has led to an increased frequency of viral infections in patients. Studies in highly heterogeneous patient populations have reported CMV infection rates ranging between 2 and 39% [1]. Chemotherapeutic agents can impair T-cell function, and the restoration of normal T-cell function may take up to a year following autologous stem cell transplantation. Patients are susceptible to viral infections during this period [2].

The most common clinical manifestation of CMV reactivation in hematological diseases after treatment is the presence of a neck mass, followed by asymptomatic imaging findings [3]. CMV reactivation can present as localized infections, such as a neck mass, or systemic diseases, such as pneumonia [4]. CMV lymphadenitis is a localized infection confined to the lymph node and may mimic relapse of hematological malignancy [5]. This report describes a patient diagnosed with Hodgkin lymphoma who developed CMV lymphadenitis mimicking lymphoma relapse one year after undergoing chemotherapy. This report primarily aims to highlight the rarity of CMV reactivation in non-transplant hematological neoplasms, the lack of specific clinical findings, and the challenges in establishing a diagnosis through laboratory or imaging studies.

## Case presentation

A 40-year-old male patient presented to our clinic in May 2022 with complaints of swelling in the neck. On physical examination, multiple firm, fixed mass lesions were detected in the right level 2-3 regions. The oropharyngeal examination was normal and endoscopic nasal examination revealed normal bilateral nasal cavities and nasopharynx. The endoscopic laryngeal examination was also normal. Ultrasonography of the neck demonstrated multiple pathological lymph nodes in both cervical jugular chains. Fine-needle aspiration biopsy (FNAB) was performed on the neck lymph nodes, and the result suggested lymphoid neoplasia. Subsequently, an excisional lymph node biopsy was performed in our clinic, and the pathology confirmed classical Hodgkin lymphoma (cHL). The patient was referred to the hematology clinic for further management.

**Figure 1:**
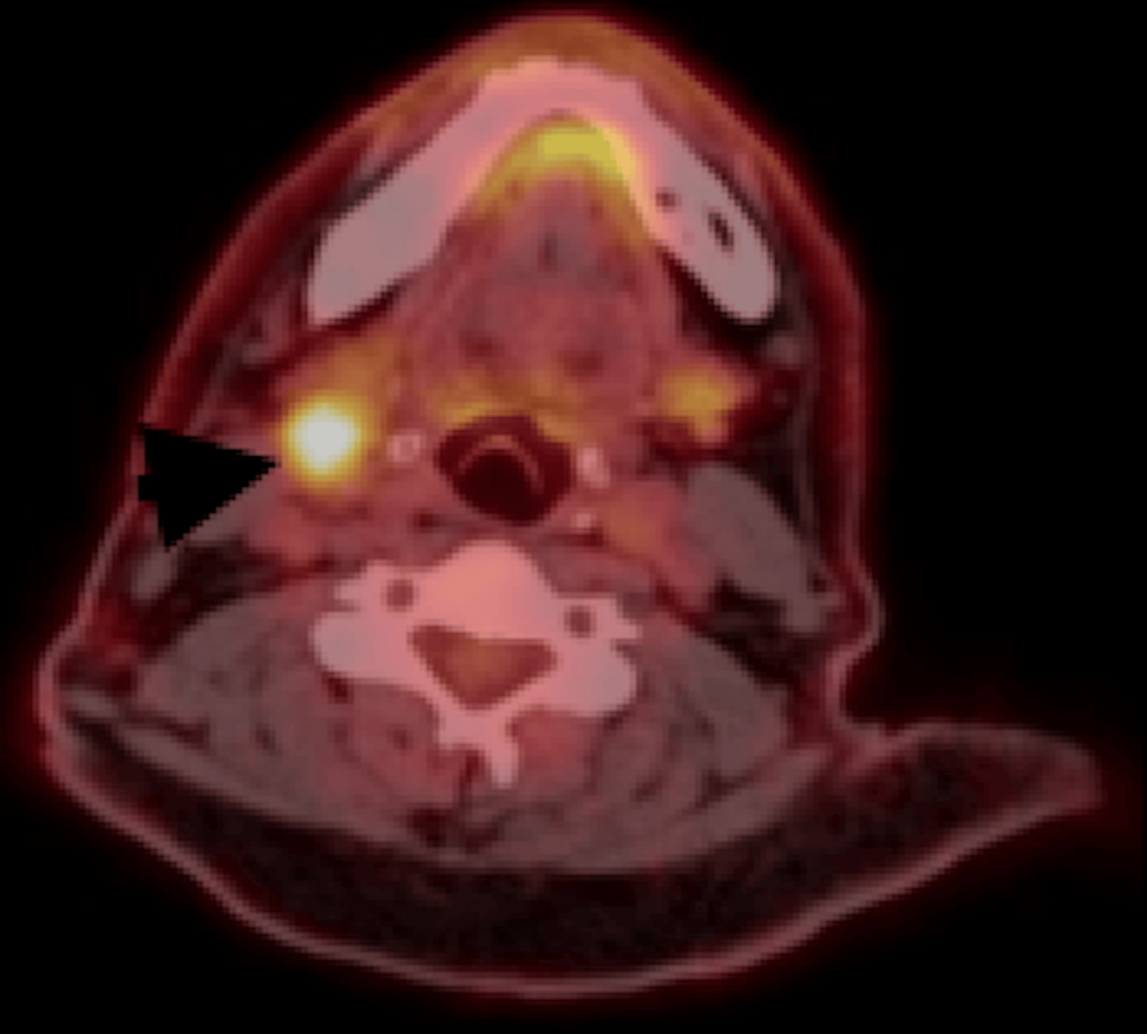
PET-CT scan findings The black arrow shows lymphadenomegaly with hypermetabolism at the right level 2 PET-CT: positron emission tomography-computed tomography

The hematology team initiated bendamustine and brentuximab treatment based on a cardiology recommendation given the patient’s history of coronary artery disease and angiography. Following eight cycles of treatment, a PET-CT scan revealed no metabolic activity, and the patient was placed under follow-up. Nine months after the completion of treatment, a PET-CT scan revealed a hypermetabolic lesion (SUVmax 19.1) in the right level 2 region of the neck (Figure 1). The patient was referred back to our clinic with suspicion of relapse or recurrence. Physical examination revealed a palpable, firm, fixed lesion at the right level 2 region, while other otolaryngological examinations were normal.

**Figure 2:**
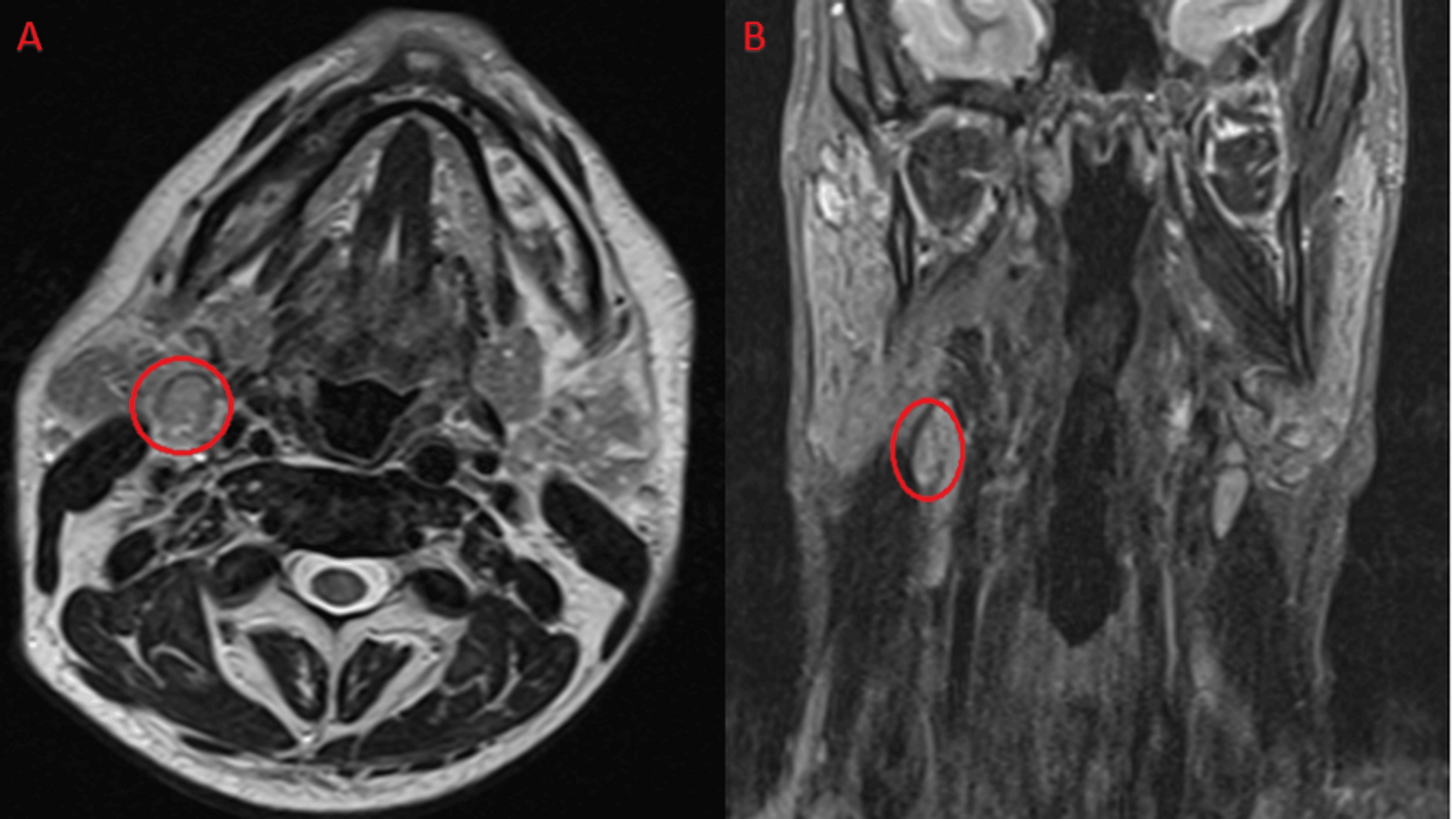
MRI of the neck (A) The red circle shows the right level 2 pathological lymph node axial section T2 sequence MRI image (B). The red circle shows the right level 2 pathological lymph node coronal section T2 sequence MRI image MRI: magnetic resonance imaging

MRI of the neck showed a pathological lymph node in the right cervical jugular chain measuring 3 cm in craniocaudal length and 1 cm in maximum diameter (Figures 2A, 2B). The hypermetabolic lesion in the right level 2 region observed on PET-CT was initially considered indicative of primary disease relapse. The Deauville score of 4 further supported the suspicion of relapse. However, the presence of isolated hypermetabolism in a single focus made recurrence less likely. Based on clinical and imaging findings, the patient underwent a repeat excisional lymph node biopsy.

**Figure 3:**
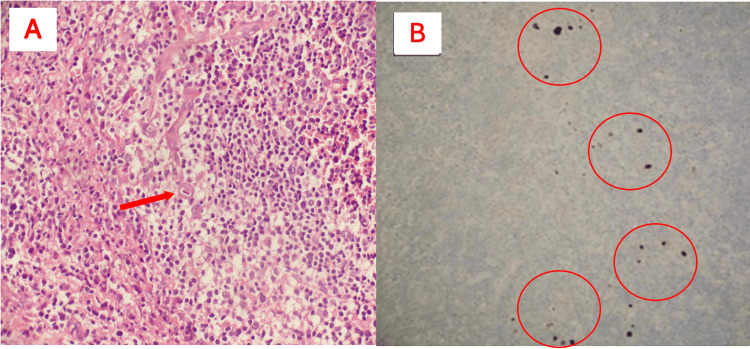
Biopsy and immunohistochemistry findings (A) Distinct immunoblasts, increased vascularity, and eosinophilic cytoplasm with pyknotic nuclei in cells of immunoblast size, observed in the hyperplastic paracortex (hematoxylin & eosin x400); the red arrow indicates the CMV viral inclusion within the cell. (B) Immunohistochemical examination revealed cells showing positive immunoreactivity for CMV (CMV x200); the red circles indicate the immunoreactive CMV inclusions CMV: cytomegalovirus

The surgical biopsy of the lymph node revealed nonspecific follicular hyperplasia. Monocytoid B-cell hyperplasia was observed in the peritrabecular and perisinusoidal areas. Additionally, large, dark, eosinophilic cytoplasmic inclusions with pyknotic nuclei, characteristic of eosinophilic viral nuclear inclusions, were identified (Figure 3A). Immunohistochemical analysis using an anti-CMV antibody revealed CMV-infected cells in some areas (Figure 3B).

Further investigations were conducted, including serum CMV PCR testing and thoracic CT. CMV DNA was not detected in the patient’s serum by PCR, and thoracic CT revealed no pathology suggestive of pneumonia. Internal medicine and ophthalmology consultations were requested to assess for systemic CMV infection and ocular involvement, respectively. The patient exhibited no signs of systemic CMV infection, colitis, or retinitis. A multidisciplinary team, including otolaryngology, infectious diseases, pathology, and hematology specialists, convened to discuss the case. Given the absence of systemic symptoms, the asymptomatic nature of the patient apart from the neck swelling, the isolated localization on PET-CT, and the patient’s comorbid conditions, it was decided to continue with monthly follow-up.

In the third month of follow-up, the patient remained asymptomatic with no pathology detected on physical examinations. Neck palpation revealed no palpable mass or lymphadenopathy. A follow-up PET-CT scan showed that the previously observed hypermetabolic focus in the right level 2 region was no longer present. Only mild hypermetabolic foci in bilateral cervical stations were detected, suggestive of benign reactive processes. The patient’s follow-up is ongoing.

## Discussion

We discuss a case of localized CMV lymphadenitis in a patient diagnosed with Hodgkin lymphoma, occurring in the ninth month of follow-up after treatment. The patient presented with neck swelling as the sole complaint. The presence of hypermetabolism on PET-CT mimicked lymphoma recurrence, necessitating a biopsy. Given that T-cell recovery after lymphoid neoplasia treatments can take over a year, infectious lymphadenitis should be considered in the differential diagnosis of these patients, as it can mimic primary malignancy recurrence.

CMV infections, particularly after lymphoma treatments and allogeneic stem cell transplantation, have been reported in the literature [6]. CMV reactivation often results in CMV pneumonia. In a case report by Delaye et al., supradiaphragmatic and infradiaphragmatic lymph nodes showing hypermetabolism on PET-CT were observed in a patient with diffuse large B-cell lymphoma who underwent allogeneic stem cell transplantation after complete remission. Lymph node sampling revealed CMV lymphadenitis. CMV IgG was positive, and CMV DNA was not detected via PCR. The patient had no systemic symptoms, indicating a localized CMV reactivation. Similarly, in our case, there was no systemic involvement, and the hypermetabolism on PET-CT prompted further evaluation. CMV IgG was positive, while blood PCR results were negative in our patient as well.

Some studies have reported that certain drugs used in chronic diseases and hematological disorders are associated with CMV reactivation. High-dose steroids, alemtuzumab, fludarabine, bortezomib, and rituximab are considered hypothetical risk factors [1]. A case report by Tudesq et al. has described CMV reactivation after brentuximab treatment, and another by Cona et al. has reported CMV reactivation following bendamustine treatment. In our case, it is noteworthy that both pharmacological agents were used in the treatment protocol [7,8].

Kang et al. reported a case of a hypermetabolic lesion in the neck six months after bone marrow transplantation, which was initially suspected to be a relapse of the primary malignancy. Histopathological diagnosis revealed CMV lymphadenitis, which spontaneously resolved without treatment. They noted that the absence of systemic symptoms and the initiation of T-cell recovery one year after primary malignancy treatment influenced the decision for close monitoring. Our case bears significant similarity to this report in terms of clinical presentation and factors influencing the decision for follow-up [9]. Another study has reported that PET-CT scans might yield false-positive results, suggesting a recurrence of the primary malignancy. In this study, lymphoma patients with Deauville scores of 4-5 comprised 30% of the cohort. In our case, the PET-CT findings were consistent with this study, as a Deauville score of 4 was reported [3].

## Conclusions

Our patient presented with enlarged cervical lymph nodes and imaging findings consistent with lymphoma recurrence, emphasizing the importance of histological examination in confirming recurrence in patients undergoing lymphoma treatment. In similar cases, the aim is to present the distinguishing features of the case, including the treatments used and the presence of a single hypermetabolic focus on PET-CT, to improve diagnostic accuracy. This case report suggests that localized CMV lymphadenitis should be considered in the differential diagnosis of these patients, even in the presence of imaging pathologies suggestive of malignancy recurrence and negative serum CMV PCR results.

## References

[1] Marchesi F, Pimpinelli F, Ensoli F, Mengarelli A (2018). Cytomegalovirus infection in hematologic malignancy settings other than the allogeneic transplant. Hematol Oncol.

[2] Delaye M, Ricard L, Maisonobe L, Chartier S, Coppo P (2022). Cytomegalovirus lymphadenitis mimicking a relapsed diffuse large B-cell lymphoma. Clin Case Rep.

[3] Yu SC, Ko KY, Teng SC (2021). A clinicopathological study of cytomegalovirus lymphadenitis and tonsillitis and their association with Epstein-Barr virus. Infect Dis Ther.

[4] Marchesi F, Pimpinelli F, Gumenyuk S (2015). Cytomegalovirus reactivation after autologous stem cell transplantation in myeloma and lymphoma patients: a single-center study. World J Transplant.

[5] Park SM, Choi YB, Lee JK (2021). Cytomegalovirus infection mimicking recurrence of malignant lymphoma: a case report. Clin Pediatr Hematol Oncol.

[6] Rossini F, Terruzzi E, Cammarota S (2005). Cytomegalovirus infection after autologous stem cell transplantation: incidence and outcome in a group of patients undergoing a surveillance program. Transpl Infect Dis.

[7] Tudesq JJ, Vincent L, Lebrun J (2017). Cytomegalovirus infection with retinitis after brentuximab vedotin treatment for CD30(+) lymphoma. Open Forum Infect Dis.

[8] Cona A, Tesoro D, Chiamenti M (2019). Disseminated cytomegalovirus disease after bendamustine: a case report and analysis of circulating B- and T-cell subsets. BMC Infect Dis.

[9] Kang KW, Lee JH, Choi JS, Lee SR, Park Y, Kim BS, Kim I (2014). Spontaneous resolution of post-transplant localized cytomegalovirus lymphadenitis mimicking tumor recurrence. Transpl Infect Dis.

